# Current Techniques and Indications for Machine Perfusion and Regional Perfusion in Deceased Donor Liver Transplantation

**DOI:** 10.1016/j.jceh.2023.101309

**Published:** 2023-11-30

**Authors:** Christopher J.E. Watson, Rohit Gaurav, Andrew J. Butler

**Affiliations:** ∗ University of Cambridge Department of Surgery, Box 210, Addenbrooke's Hospital, Cambridge, CB2 2QQ, UK; † The Roy Calne Transplant Unit, Addenbrooke's Hospital, Cambridge, CB2 2QQ, UK

**Keywords:** liver preservation, ischemia reperfusion injury, machine perfusion, donation after circulatory death

## Abstract

Since the advent of University of Wisconsin preservation solution in the 1980s, clinicians have learned to work within its confines. While affording improved outcomes, considerable limitations still exist and contribute to the large number of livers that go unused each year, often for fear they may never work. The last 10 years have seen the widespread availability of new perfusion modalities which provide an opportunity for assessing organ viability and prolonged organ storage. This review will discuss the role of *in situ* normothermic regional perfusion for livers donated after circulatory death. It will also describe the different modalities of *ex situ* perfusion, both normothermic and hypothermic, and discuss how they are thought to work and the opportunities afforded by them.

## Graphical abstract

**Figure:**
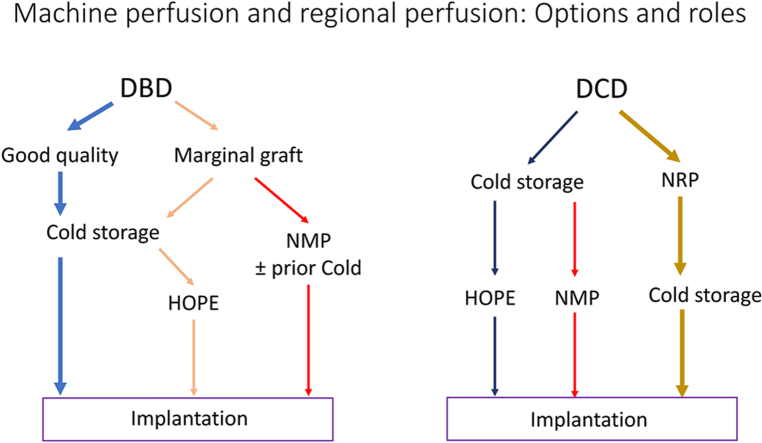

The 2021 report of the Organ Procurement and Transplantation Network/Scientific Registry of Transplant Recipients (OPTN/SRTR) details transplant activity in the USA. It records 10% of the livers recovered from the 9540 “liver donors” going unused, an increase from 8.4% in 2018.^1^ Common reasons cited include donation after circulatory death, older donors, and livers with a risk of disease transmission such as hepatitis C. Interestingly, OPTN/SRTR also report that there were actually 13,862 deceased donors in 2021,^2^ implying that 4322 (31%) donors were not considered to be liver donors. Meanwhile 1134 patients died while waiting, and 1177 became too sick to transplant. Many more that might have benefitted may not have had access to liver waiting lists.

This pattern of deceased donor liver utilisation is not unique to the United States. Similar data exist for the United Kingdom, elsewhere in Europe, and elsewhere in the world. It reflects biases built up over time, some evidence-based others not, on what may or may not be not a viable liver, and what constitutes a suitable liver donor. They reflect an era when viability was assessed based on the medical history of the donor and their biochemistry before donation, with no measure of function post organ recovery prior to implantation. They also reflect increasing external scrutiny of transplant programmes which encourages conservative practices.^3^, ^4^, ^5^, ^6^ The data also reflect a human element – no surgeon wants to make the wrong decision and be directly responsible for the death of their patient. If the patient were to die on the waiting list, the surgeon may see that more as an act of God, rather than reflecting their own conservative implantation practices; an act of commission is harder to justify than an act of omission.

Table 1 highlights some of the factors identified by some analyses of transplant registries that predict outcomes post-transplant, and which influence decision making. Other factors have also been identified that have not been incorporated into the indices, such as extraction time and anastomosis time.^7^ In addition, some factors are not significant because livers with such factors are simply not used. One example is asystolic time, the time from circulatory arrest to cold *in situ* perfusion. An unpublished analysis of the UK Transplant Registry showed that asystolic time had no bearing on the outcomes of liver transplantation, probably because livers with extremes of asystolic time were not transplanted.

**Table 1:** Adverse Factors Identified in Risk Prediction Scores.

	Factor	US DRI Feng^141^	UK DLI Collett^142^	ID2EAL Asrani^143^	Machine learning. Lau^144^	UK DCD risk, Schlegal^145^	Eurotransplant marginal criteria^146^
**Donor factors**	DCD	x	x	x	x	x	x
	Age	x	x	x	x	x	x
	Cause of death	x		x	x		
	Height	x	x	x			
	Weight			x			
	Body mass index					x	x
	Sex		x				
	Race	x					
	Smoker		x				
	Insulin-dependent diabetic			x	x		
	Alcohol consumption				x		
	Previous abdominal surgery				x		
	Distance from recipient	x			x		
	ICU stay >7days						x
	ALT						x
	AST						x
	Bilirubin		x				x
	Creatinine			x			
	Sodium						x
	Albumin/protein				x		
	Hb				x		
	CMV status				x		
	HSV antibodies				x		
	Steatotic appearance						x
	Pancreas retrieval				x		
	Split	x	x				
	WIT/FWIT					x	
	CIT	x				x	
**Recipient**	Retransplant					x	
	MELD-Na					x	
	Age					x	
	Hospitalised or home				x		

ALT: alanine transaminase; AST: aspartate transaminase; CIT: cold ischemic time; CMV: cytomegalovirus; DCD: Donation after circulatory death; Hb: Hemoglobin; HSV: Herpes simplex virus; MELD-Na: Model for end-stage liver disease-sodium; WIT/FWIT: (Functional) warm ischemic time

**Table 2:** Summary of Novel Perfusion Techniques.

Technique	Advantages	Disadvantages	Key evidence
*In situ* normothermic regional perfusion (NRP)	• Treats all organs being donated • Restores intracellular energy stores before period of cold ischaemia • Associated with low rate of cholangiopathy, probably by flushing out accumulating fibrin or fibrinolysis • Relatively cheap, compared to NMP	• Requires additional perfusionist and extracorporeal circuit	Hessheimer *et al.*^31^ Jochmanns *et al.*^38^ Watson *et al.*^32^
Hypothermic oxygenated perfusion (HOPE)	• Performed during hypothermia • Simple to perform • Minimal requirement for continued supervision • Associated with low rate of cholangiopathy	• Currently available equipment is not portable • Requires instrumentation of portal vein (and hepatic artery if dual HOPE) • Cannot assess viability, but rather it quantifies damage	Van Rijn *et al.*^52^ Schlegal *et al.*^54^
Controlled oxygenated rewarming (COR)	• Associated with less reperfusion injury • Role is probably as a component of HOPE	• Experimental work has concentrated on COR from 4 °C to 20 °C	Minor *et al.*^56^ Hoyer *et al.*^57^
Persufflation	• Simple and cheap • Regenerates ATP stores	• Long history, but has failed to translate into clinical reality • Disappointing clinical trial results	Gallinat *et al.*^72^
Normothermic machine perfusion (NMP)	• Enables assessment of graft function, as well as graft damage • Facilitates prolonged extra-corporeal storage • Fully automated machines available • Better early graft function post implant • Addition of TPA and FFP facilitates the clearance of the fibrin that causes cholangiopathy	• Expensive consumables • Normothermia means that should the circuit fail, the liver is lost	Nasralla *et al.*^84^ Markman *et al.*^85^ Watson *et al.*^130^

Recent developments in organ recovery and preservation are set to change the way livers are considered for transplantation. They hold the promise of reversing ischaemic injury, minimising reperfusion injury, and manipulating previously undesirable livers such as those with steatosis or from donation after circulatory death (DCD) donors, making them more acceptable prospects for implantation. In this paper we will discuss novel methods of organ preservation (Table 2), and identify ways in which they may be used to improve the availability and function of livers for transplantation, and facilitate more appropriate liver selection for recipients,^8^ relying less on belief and guesstimated outcome and more on direct assessment. These developments may improve access to liver transplantation, allowing a change in the current restrictive patient selection processes.

## Ischaemia

Since the inception of transplantation there has been an obligate period of ischaemia as the liver is removed from the donor, and of reperfusion injury as a supply of oxygenated blood is restored. With the exception of the pioneering work of Guo *et al.*,^9^ that remains the case today. Ischaemia is characterised by a number of metabolic processes that have been elegantly described elsewhere, but which bear repeating in order to understand the challenges and benefits of novel preservation techniques.

**Figure 1:**
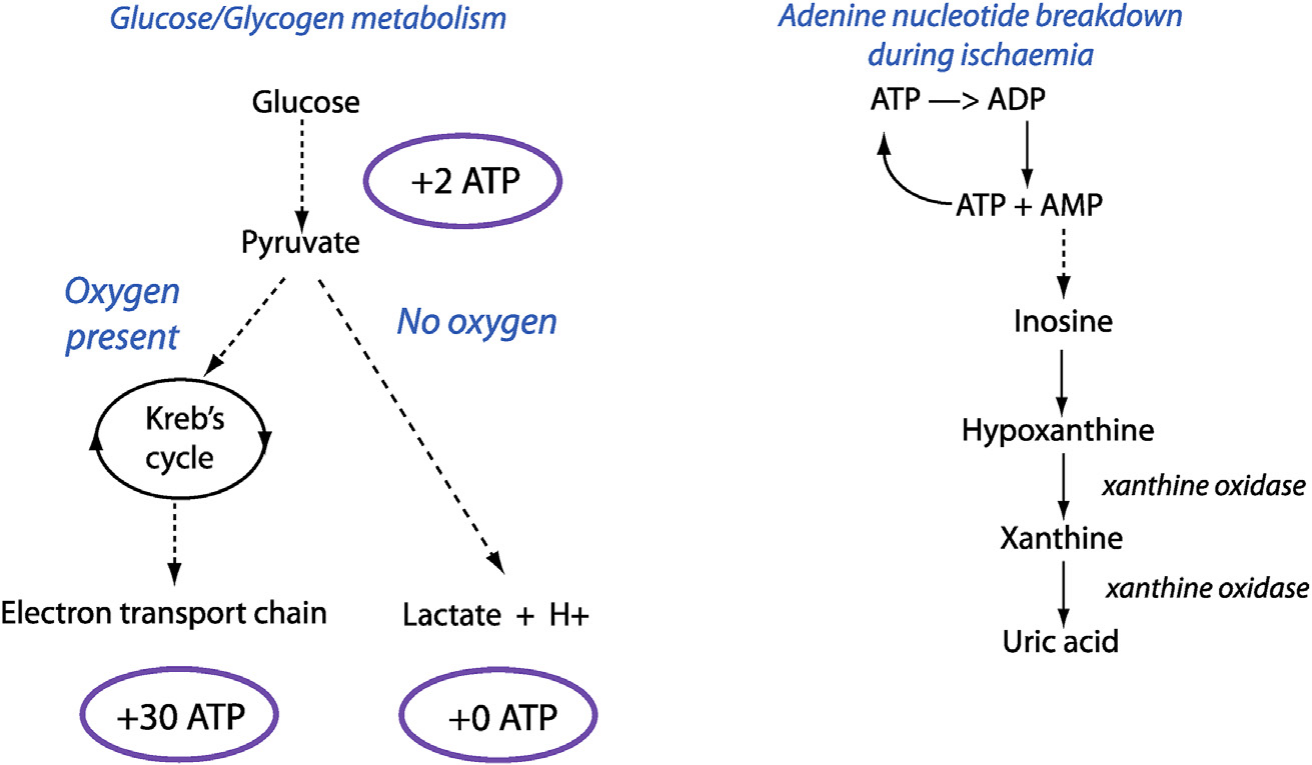
**Generation of ATP during ischaemia**. Glycogen is converted to glucose-6 phosphate and thence to pyruvate. In the absence of oxygen, this is ultimately metabolised to lactate. When oxygen is present, pyruvate is able to generate more ATP via the citric acid (Krebs) cycle and electron transport chain of the mitochondria. As ATP depletes, it starts to be degraded first to ADP, then AMP, and finally uric acid.

In order to remain viable, cells need to retain their supply of energy, in the form of adenosine triphosphate (ATP). Cooling the organ slows metabolism and the consumption of ATP. Nevertheless, metabolism continues and in the absence of oxygen the cell switches to anaerobic metabolism. Glucose, obtained by the conversion of glycogen, is consumed and ATP produced, but 16 times less efficiently than in the presence of oxygen (Figure 1).^10^ Lactic acid accumulates as a consequence, and inhibits further glycolysis. In order to generate ATP when glucose is depleted, accumulating ADP is converted to ATP and AMP (2ADP = ATP + AMP), and the accumulating AMP is deaminated to inosine monophosphate (IMP), and thence to hypoxanthine and xanthine (Figure 1).^10^ Preservation solutions like the University of Wisconsin solution aim to preserve adenosine substrates by including allopurinol to block xanthine oxidase and a buffer to counter the accumulating protons.

The catabolic state that characterises the critically ill donor, and the practice of withholding supplementary parenteral nutrition for the first 7–10 days if enteral nutrition is not tolerated,^11^ depletes glycogen stores within the liver before donation. In the DCD donor, this is further compounded by the catecholamine release following withdrawal of treatment that drives further glycogenolysis before cold storage begins.^12^ The end result is a donor liver depleted of the necessary energy substrates to tolerate long periods of ischaemia. This is most marked in DCD donor livers and livers from donors with prolonged inpatient stays, both identified previously as risk factors for poor function (Table 1).

## Reperfusion injury

It is now established that during ischaemia, succinate accumulates within mitochondria. On reperfusion the accumulated succinate is rapidly oxidised causing generation of reactive oxygen species by reverse electron transport.^13^^,^^14^ This results in mitochondrial and hepatocellular disruption. This process complements and further enhances the inflammatory response that characterises reperfusion injury, with release of damage-associated molecular patterns (DAMPS), chemokines, and pro-inflammatory cytokines stimulating infiltration of immune cells.^15^, ^16^, ^17^ In addition, in the absence of adenosine substrates, resumption of ATP generation may take some time, and may account for delayed fall in lactate observed during some dynamic perfusion techniques.

## *In situ* normothermic regional perfusion (NRP)

### History

*In situ* oxygenated perfusion of deceased donors was begun as a means to cool the donor prior to organ recovery, but this has been superseded by normothermic perfusion which affords an opportunity to assess organs and extend preservation. Core cooling began in Japan,^18^^,^^19^ and was also undertaken in Taiwan as a means of extending preservation pending permission to recover organs for transplantation.^20^

*In situ* normothermic regional perfusion (NRP) was developed in Barcelona for the recovery of organs from uncontrolled DCD (uDCD) donors,^21^^,^^22^ but has become established as the preferred method for recovery of organs from controlled DCD donors;^23^, ^24^, ^25^ in France it has been mandatory to use NRP for the recovery of livers since the inception of their controlled DCD liver transplant programme.^26^

### Technique

**Figure 2:**
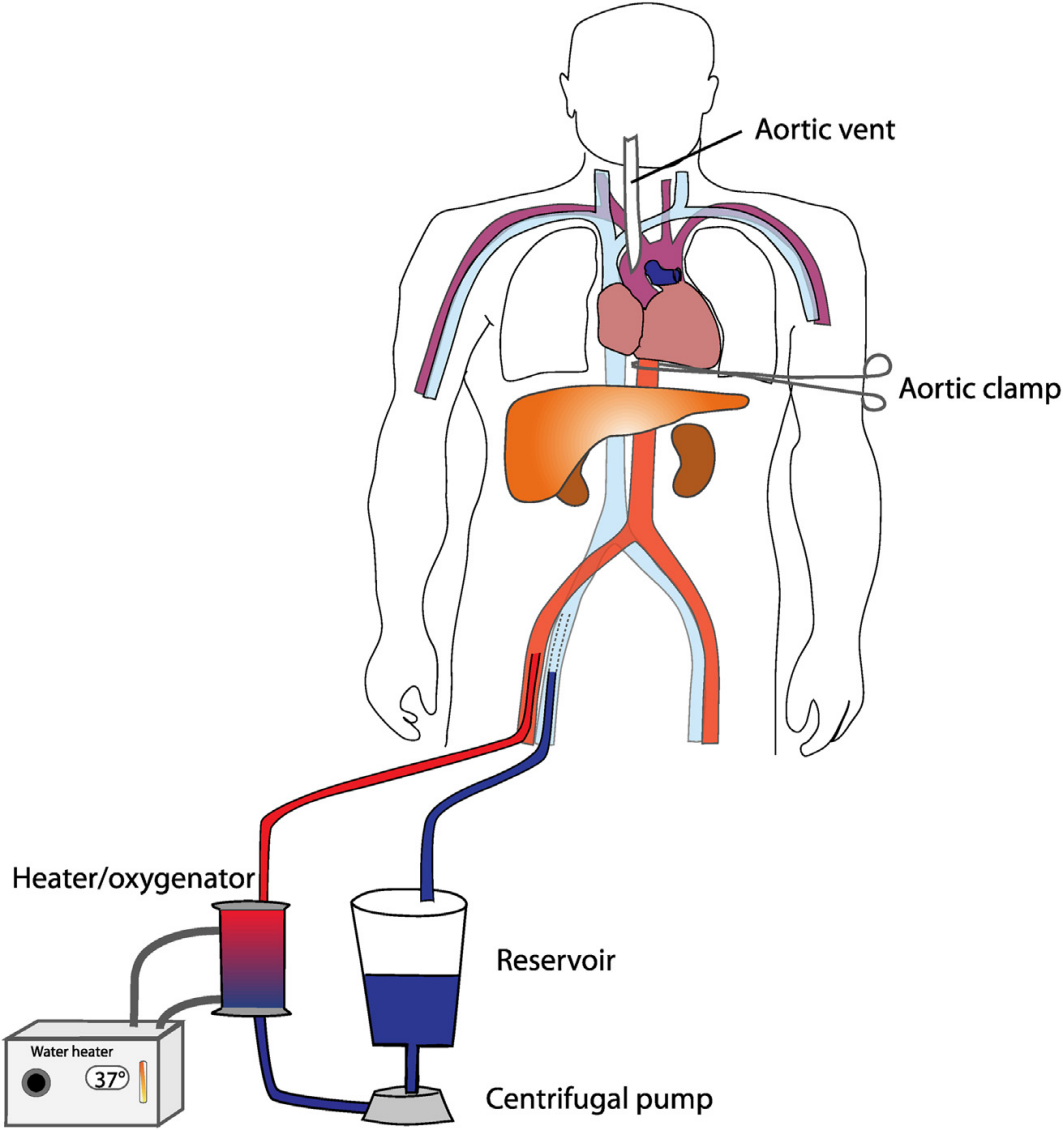
**Normothermic regional perfusion**. Donor venous blood is pumped through an oxygenator/heater and returned to the arterial circulation. It is prevented from perfusing the brain by the presence of a cross-clamp across the descending thoracic aorta. A vent in the ascending aorta verifies that there is no perfusion of the brain.

NRP involves restoring circulation of blood to the abdominal organs while ensuring no perfusion of the brain. This is achieved using a modified extracorporeal oxygenation circuit comprising pump, membrane oxygenator and heater (Figure 2). Where local laws permit, the femoral vessels are cannulated pre-mortem and/or heparin is administered to the donor. In other countries, such as the UK, no pre-mortem intervention is permissible; in that case cannulation of femoral vessels, or IVC and abdominal aorta, takes place after death, and large doses of heparin are delivered on commencement of perfusion. Following death and cannulation, the circuit is established, de-oxygenated blood is drained from the donor's venous system and oxygenated blood returned to the arterial circulation. A cross clamp across, or endoluminal occlusion balloon within, the descending thoracic aorta limits the organs being perfused, and a vent in the ascending aorta ensures no perfusion of the brain.^27^ Alternatively, the branches of the aortic arch to the head and upper limbs may be clamped and divided, and the restored circulation may be used to supply blood to the coronary arteries and to restart the heart. Hearts recovered in this manner can function as well as hearts from DBD donors.^28^

NRP is usually instituted in the operating theatre, and withdrawal of treatment is preferably in the same location. NRP may be established by the retrieval team, or by a mobile unit whose role is only to establish donors on NRP while leaving a separate team of retrieval surgeons to retrieve the organs. In France, NRP is often instituted in the intensive care unit, and the donor transferred to the operating theatre on NRP once the family has seen the donor one final time.

NRP is usually run for 1–4 h,^29^ although this has been extended further in Italy,^30^ where the requirements for the certification of death require a 20 min “no-touch” period of absent electrical activity. As a consequence, in Italy, most livers also undergo a period *ex situ* perfusion following NRP.

### Functional Assessment

One of the advantages of NRP is that it allows functional assessment of the liver. Function can be inferred from minimal release of transaminases into the circulation, or from the clearance of lactate. Transaminase release, specifically alanine transaminase (ALT), is a common marker which can be measured using a near-patient device. ALT is generally preferred to aspartate transaminase (AST) since it is liver specific, while AST also exists in erythrocytes and other organs including the heart. Initial uncontrolled DCD work in Barcelona accepted livers providing the transaminases rose to less than 4x the upper limit of normal, while UK practice currently accepts a 10-fold rise, providing excess transfusion was not required to maintain the NRP circuit. Lactate falls during NRP, but seldom to normal levels since there remains lactate rich blood returning to the circuit from non-perfused areas of the body. The other monitoring required is the venous oxygen saturation (SvO_2_), which should be maintained between 60 and 80 % to ensure adequate oxygenation. A fall in SvO_2_ is corrected by optimising oxygen administration, increasing the perfusion flow rates, and/or increasing oxygen carriage by adding more blood. There are currently no evidence-based criteria that determine viability during NRP.

### Clinical Evidence

Although there are no randomised trials of NRP, the evidence to date is overwhelming that a period of NRP before recovery of the organs improves the outcomes of livers and kidneys, and reduces the incidence of biliary complications compared to traditional rapid recovery techniques.^25^^,^^31^, ^32^, ^33^, ^34^ Indeed, many authors have likened a liver from an NRP DCD donor to one donated after brain death.^26^ The enhanced ischaemic time tolerance and superior outcome of NRP livers are difficult to explain, but one suggestion is that NRP may provide an ischaemic preconditioning stimulus which enhances the tolerance to further periods of ischaemia.^35^ It has also been shown to facilitate ATP generation prior to cold storage, replacing the ATP that has been depleted during warm ischaemia.

Not only does NRP improve the outcomes of the livers, but there is increasing evidence that it facilitates utilisation of livers that would previously have not been considered suitable.^36^^,^^37^ Recent consensus guidelines from the Europan Society for Organ Transplantation recommended NRP for both uncontrolled and controlled DCD donation.^38^

## *Ex situ* machine perfusion (ESMP)

In contrast to *in situ* regional perfusion, which is almost universally managed at normothermia, several different modalities of ESMP exist. *Ex situ* and *in situ* are the preferred descriptive terms when discussing perfusion, since NRP occurs after death (*ex vivo*) as does isolated organ perfusion.

## Hypothermic machine perfusion (HMP)

Early studies showed that simple non-oxygenated hypothermic machine perfusion (HMP), applied after a period of static cold storage and before implantation (so called end-ischaemic perfusion^39^) could facilitate the transplantation of livers rejected by others.^40^ The benefit of such perfusion may reflect the ability to remove accumulating lactate and to provide adenosine substrate to hepatocytes, as well as flushing the vessels of accumulated fibrin. Guarrera *et al.*^40^ also used a modified perfusion fluid, comprising Vasosol with added l-arginine, n-acetylcysteine, nitroglycerin, and prostaglandin E1. Vasosol itself contains a buffer, an impermeant, and adenine as a substrate for ATP generation. It is not clear which, if any, of these is important. Analysis of liver biopsies suggested a reduction in the inflammatory markers of ischaemia and reperfusion following HMP.^41^

## Hypothermic oxygenated perfusion (HOPE)

### Background

**Figure 3:**
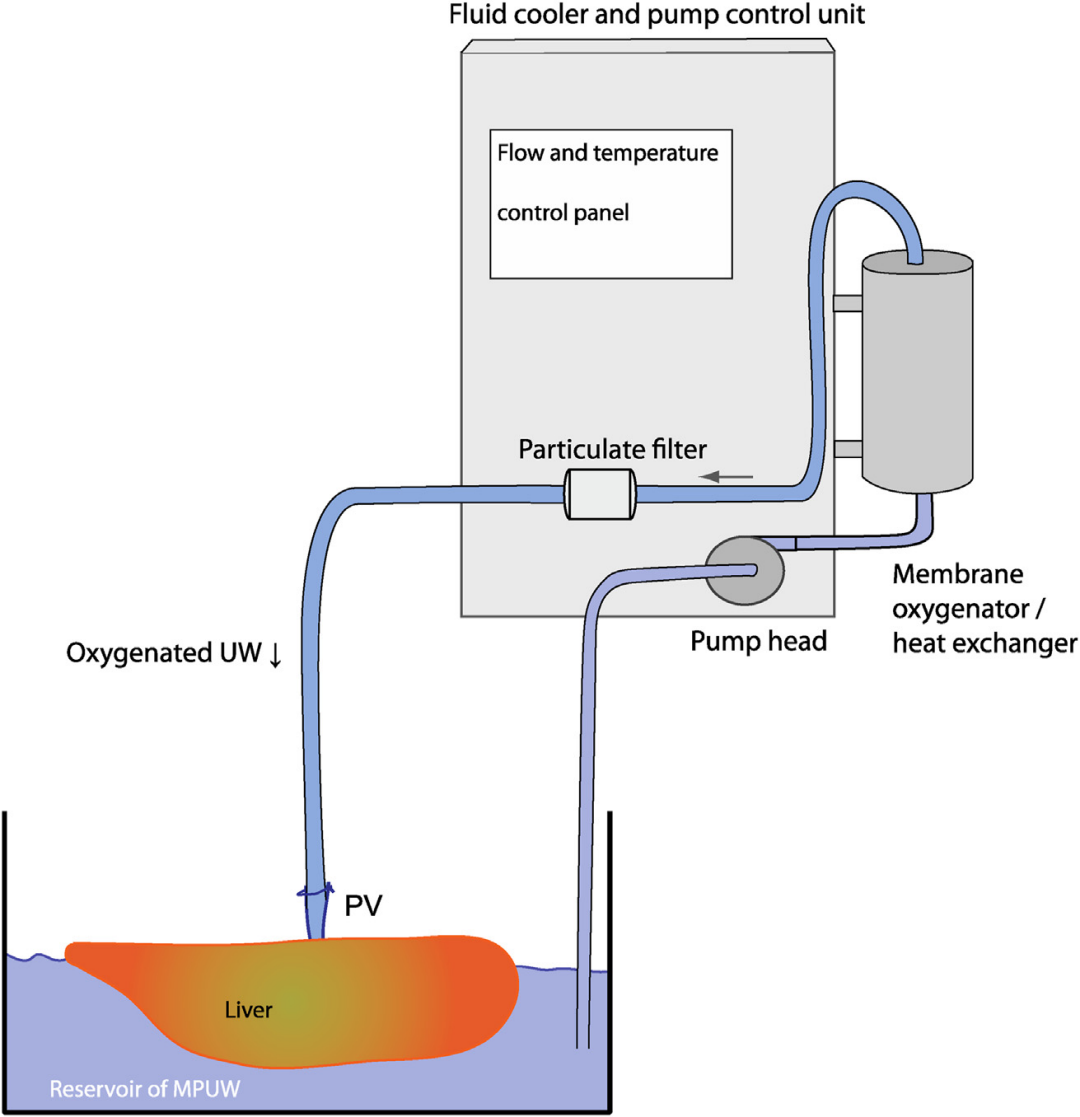
**Hypothermic oxygenated perfusion (HOPE) of the liver**. During hypothermic oxygenated perfusion, the liver rests in a bath of cold Machine Perfusion University of Wisconsin solution (MPUW) and this is recirculated and oxygenated. HOPE perfuses just the portal vein, whereas D-HOPE perfuses both portal vein and hepatic artery. The recirculated fluid passes through a particulate filter before return to the liver. There is no clear benefit of dual perfusion over single perfusion.

While Guarrera *et al.*were developing hypothermic perfusion, Dutkowski et al.in Zurich were oxygenating ice cold Machine Perfusion University of Wisconsin (MPUW) solution and circulating it through livers (Figure 3). Experimentally, they showed that sufficient oxygen was carried at low temperatures to enable repletion of ATP,^42^ and that it was effective for preventing some of the reperfusion injury exhibited by DCD livers.^43^ They went on to show its efficacy in clinical transplantation with DCD donors with prolonged asystolic warm times which characterise DCD donation in Switzerland.^44^ The ability to moderate reperfusion injury probably reflects both the relatively low oxygen carriage in perfusate in the absence of erythrocytes, and the fact that cold will reduce the rate of succinate dissipation and consequent ROS production.

The attraction of HOPE, like HMP, is its simplicity. It may be undertaken with bespoke machines or with locally adapted cardiopulmonary bypass or ECMO equipment. While it was originally conducted with the liver in a bowl of ice cold UW solution, the Liver Assist device (XVIVO, Groningen, NL) runs HOPE at 10 °C, which is the lowest temperature it can achieve. There are also two different approaches to cannulation. Dutkowski's original studies perfused the liver through the portal vein alone, while Porte adapted Dutkowski's HOPE principle but perfused both artery and portal vein, and termed it Dual HOPE (D-HOPE).^45^ The benefits of the portal only approach would be to ensure any fibrin or other intra-arterial material would be flushed retrogradely out of the artery during perfusion, as opposed to the D-HOPE approach which would flush it into the capillaries and peribiliary plexus. There appears to be little real difference in hepatocyte or endothelial preservation between the two techniques.^46^^,^^47^ In this review, we have considered them both the same.

Originally HOPE was undertaken in the hour or two before the liver was implanted.^48^ A recent publication describes a DBD liver preserved for 11 h using HOPE,^49^ and extended preservation is currently the subject of a clinical trial in Europe.^50^

### Functional Assessment

Unlike NRP and normothermic machine perfusion (NMP), there is no ability to test liver function during HOPE (or HMP). The Zurich group has proposed measuring flavin mononucleotide (FMN) dynamically to give an indication of viability.^51^ FMN is a component of complex 1 of the mitochondrial respiratory chain. Complex 1 injury, as occurs when succinate is rapidly metabolised by succinate dehydrogenase when reoxygenation occurs, generates ROS and results in mitochondrial and cellular damage, with release of FMN into the perfusate.^14^ FMN itself can be colorimetrically detected real time in perfusate fluid. As might be expected, there is a highly significant correlation between FMN release and ALT release into the perfusate, since both are cell injury markers.^51^ There is also a correlation of FMN with lactate post-transplant, probably reflecting the capacity of the remaining intact hepatocytes. Hence a damage marker like FMN and ALT gives an indirect readout on functional capacity, not only in HOPE but also in NRP and NMP.

There is an additional benefit of HOPE proposed in the treatment of steatotic livers. Such livers are very prone to lipid peroxidation as a result of ROS generation. In addition, traditional cold storage temperatures of around 4 °C result in change in phase of accumulated intracellular lipid, with resultant disruption of some hepatocytes. Reperfusing livers at 10 °C may facilitate clearance of some of the extravasated lipid, and open up vascular spaces previously occluded by the intravascular debris and swollen hepatocytes. This is in contrast to normothermic perfusion, where the liver beyond the occluded vessels will suffer warm ischaemic damage and necrosis; in the cold this period of hypoperfusion is tolerated until perfusion is re-established.

### Clinical Evidence

A European randomised trial compared HOPE with static cold storage (SCS) in DCD liver transplantation and showed a reduction in post reperfusion syndrome (12 % vs. 27 %) and in early allograft dysfunction (26 % vs. 40 %),^52^ although the latter, based as it is largely on transaminase release,^53^ is difficult to interpret in a liver that has been perfused *ex situ* since the act of perfusion washes out ALT and will result in a lower level posttransplant. Most important was a reduction in the incidence of “clinically significant” non-anastomotic biliary strictures at 3 months (6 % vs. 18 %), although there was no difference in the incidence of any non-anastomotic stricture (62 % HOPE vs. 58 % SCS).^52^ There was a similar incidence of anastomotic biliary stricture (29 % HOPE vs. 28 % SCS) and leakage (8 % HOPE vs. 10 % SCS), and no difference in 6 month graft survival. The persistence of non-anastomotic biliary strictures, albeit not as clinically significant in other series, was disappointing. Longer term follow-up of this study is awaited to see how the strictures that were deemed not to be clinically significant evolve.

A second European randomised trial studied HOPE in DBD livers, and randomised 177 livers resulting in 85 HOPE transplants and 85 SCS transplants.^54^ The main end point of the study was the incidence of complications according to the Clavien-Dindo scale (Clavien was also a co-author of the HOPE study).^55^ There was an equal proportion of livers experiencing a Clavien ≥3 complication in each group (51.8 % HOPE vs. 54.1 % in SCS; OR 0.91, *P* = 0.76). Post hoc analyses suggested there were more severe complications (Clavien ≥3b) in the SCS group (RR 0.26, *P* = 0.027). Aside from these post hoc analyses, the study was disappointing. There was no significant difference in post-transplant chemistry in the first week (as assessed by area under the concentration time curve) although there was a numerical difference in early allograft dysfunction (45.9 % SCS, 16.5 % HOPE) as might have been predicted due to transaminase washout (see above). There was no difference in ICU and total hospital stay, and no significant difference in graft survival at 12 months (95.3 % HOPE, 91.8 % SCS). Four patients in each group died in the first year.

## Controlled oxygenated rewarming (COR)

Another technique proposed to improve outcomes of livers that have been cold stored is controlled oxygenated rewarming (COR). This draws on the observation that a period of oxygenated cold perfusion can restore ATP with minimal ROS generation, and acknowledges the necessary change in physical state of lipid membranes as organs transition from being stored on ice to being reperfused with blood at 37 °C. It allows the organ to recover metabolism slowly, in contrast to reperfusing with normothermic blood which challenges the organ to move immediately from minimal metabolism to maximal metabolism to repair the energy-depleted organ. Initial pig work showed that a 90 min period of COR, raising the temperature of an SCS liver to 20 °C before transplantation, resulted in regeneration of ATP, with minimal generation of oxygen-free radicals comparted to livers subjected to SCS alone.^56^ This resulted in lower levels of transaminase release, signifying less hepatocellular injury.

### Clinical Evidence

An initial study of six livers allocated by Eurotransplant's “Rescue allocation” was compared to 106 controls and showed similar benefits to those seen in the pig work, with lower release of transaminase and better early function.^57^ The group went on to undertake a randomised single centre trial with 20 COR transplants compared to 20 with SCS alone, using the Liver Assist (XVIVO, Groningen) device to achieve the controlled oxygenated rewarming. This small trial demonstrated less transaminase release, a significantly lower day 7 AST and bilirubin, and better function as assessed by the LiMax test on day 1. There were twice as many Clavien–Dindo ≥3b complications in the control group than the COR group. At the end of a year 100 % of the COR grafts were still functioning, compared to 95 % of the controls (p = n.s.).

The relative benefits of sustained periods of HOPE compared to SCS followed by pre-implant COR remain to be explored.

## Persufflation

The final technique of hypothermic storage that will be discussed is persufflation. This is an old technique, first discovered by accident over 100 years ago, which involves passing oxygen (persufflating) through the vascular tree of an organ in the cold.^58^ Its use was initially explored in cardiac surgery,^59^, ^60^, ^61^ and later in transplantation, and in particular kidney transplantation where oxygen is passed up the renal vein and escapes through a series of pinpricks in the capsule of the kidney.^62^ It is believed that its main benefit is to reduce the loss of ATP during ischaemia, with reduced consumption of glycogen and glucose stores.^63^, ^64^, ^65^ Livers and hearts have also been shown to tolerate extracorporeal storage better when subject to persufflation,^66^, ^67^, ^68^, ^69^ and to increase ATP stores when administered end-ischaemic.^70^ In contrast to HMP, HOPE and COR, no additional period of machine perfusion is required during persufflation.

### Clinical Evidence

A pilot study demonstrated the feasibility of the technique in the clinical setting by persufflating five marginal livers. All five had good early function and maintained function for at least 2 years.^71^ The same group from Essen then undertook a randomised controlled trial randomising 116 subjects, with 57 receiving persufflated livers.^72^ Persufflation was performed during the pre-implant bench work with the liver on ice and a catheter inserted into the vena cava, which was closed at one end to ensure retrograde gas passage. Small pinpricks were performed on the liver capsule to allow gas to escape, and persufflation continued for at least 2 h. There was no significant difference in transaminase release or early function, although the median peak AST was lower in the persufflation group (median 972iu/L, range 194-17577iu/L, in the persufflation group; median 1246iu/L, range 310-8064iu/L, in the control group) and no difference in the 5-year graft and patient survival, which were confounded by the incidence of recurrent hepatitis C and hepatocellular carcinoma in the recipients.

## Normothermic machine perfusion

**Figure 4:**
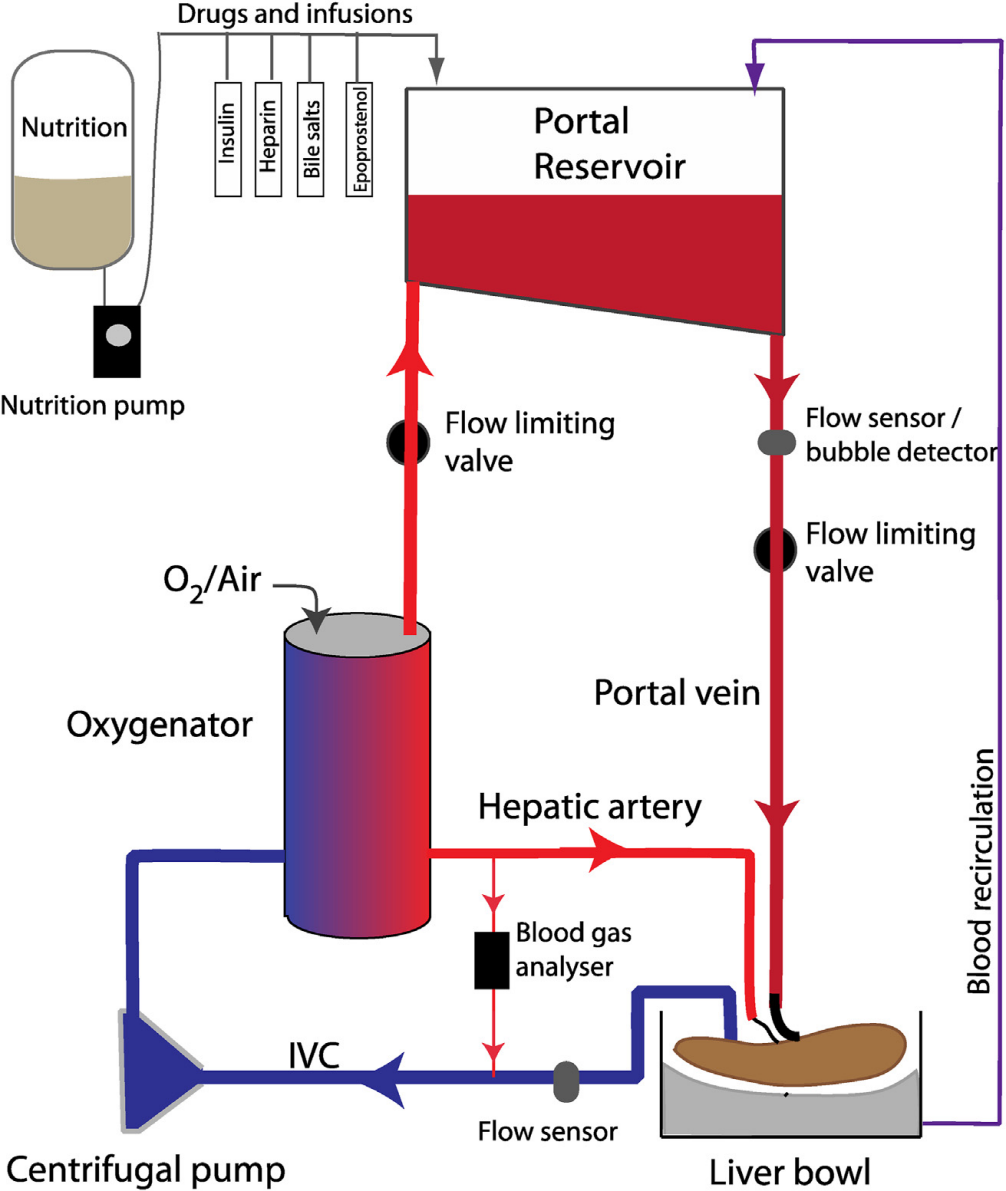
**Normothermic machine perfusion of the liver**. During normothermic liver perfusion, the liver receives a supply of oxygenated blood via both artery and portal vein; the latter may either be via dependent flow as illustrated here, or directly pumped. Effluent blood then passes back via a venous cannula to be pumped around the circuit once again. During perfusion the liver receives a number of reagents designed to improve perfusion, such as insulin and epoprostenol as well as nutrition.

Normothermic machine perfusion (NMP, Figure 4) aims to restore normal function to the liver to enable extended preservation and assessment of suitability for transplantation, while doing so in an environment that minimises reperfusion injury. Early animal work showed the feasibility of isolated liver perfusion and was mostly used to study liver physiology and function,^73^ but it was the advent of cheaper disposable cardiopulmonary bypass components that made clinical normothermic liver perfusion a possibility.

One of the first uses of NMP was to support patients in fulminant liver failure pending recovery of their own liver or as a bridge to transplantation.^74^ Around the same time the first animal transplants of livers preserved by NMP were being performed, which showed superior preservation to cold stored livers.^75^ Subsequent e*x situ* assessment also showed the superiority of NMP over SCS.^76^ The ability to safely extend preservation to 72 h was also demonstrated at that time.^77^

### Technique

There are two types of devices currently available for liver NMP, depending upon whether the perfusion circuit is open to air or closed. Currently the OrganOx *metra* and the Transmedics Liver OCS devices are closed circuits and the XVIVO Liver Assist is an open circuit. For closed circuits, the hepatic artery, portal vein, and IVC are cannulated, with the suprahepatic IVC being oversewn or stapled. Blood in the IVC is removed under negative pressure, and circulated through a heater/oxygenator before being returned to the hepatic artery and portal vein. Any blood leaking from small vessels other than the named vessels is collected in a reservoir and recirculated into a reservoir. For open circuits, the liver is suspended over a reservoir and blood from the IVC flows directly into the reservoir; the IVC is not cannulated and remains open at both ends. The hepatic artery and portal vein are cannulated for inflow. The other difference between devices is whether the portal pressure is achieved by having the portal reservoir suspended above the liver, creating a hydrostatic pressure, or whether the portal blood is actively pumped into the liver. Where blood is pumped into the liver, whether artery or vein, it is regulated by pressure sensors on the inflow cannulae. It has been shown that epoprostenol infusion is associated with superior perfusion of the liver,^78^ and infusion of bile salts results in better preservation of the biliary tree.^79^

The Liver Assist is an ideal device for research, since every element is modifiable; in contrast, the OrganOx and Transmedics devices are fully automated and able to be transported with the liver on board, and are much simpler to manage for clinical purposes.

### Clinical Evidence

The first clinical liver transplantation following NMP took place in London in 2014, but was not reported until 2016 ^80^; meanwhile other case reports and series were published from Europe and North America.^81^, ^82^, ^83^ The initial pilot series involved 20 NMP liver transplants perfused on the OrganOx *metra*, compared to 40 historic SCS controls; livers were placed on NMP at the donor hospital. As with other perfusion studies, peak transaminase in the first 7 days was less with NMP, and the study showed the feasibility of NMP.^80^

A subsequent European randomised trial, with peak transaminase as the primary endpoint, randomised 335 livers of which 222 were eventually transplanted, 121 in the NMP arm and 101 in the SCS arm, reflecting the ability of NMP to increase utilisation.^84^ As with the pilot studies, livers were placed on NMP at the donor centre. There was a significant benefit of NMP in lowering post-transplant transaminase release, with a bigger effect seen in DCD livers than DBD livers. Post reperfusion syndrome was reduced (12.4 % NMP vs. 33.0 % SCS), and early allograft dysfunction was also reduced (10.1 % NMP, 29.9 % SCS). There was no significant difference in the incidence of non-anastomotic biliary strictures (DCD livers: 11.1 % NMP vs. 26.3 % SCS, *P* = 0.180; DBD livers: 7.4 % NMP vs 5.4 % SCS, *P* = 0.678). There was no difference in graft or patient survival.

A subsequent trial undertaken in the US using the Transmedics Organ Care Systems (OCS) Liver device used the incidence of early allograft function as a primary endpoint.^85^ As with the OrganOx *metra* study, livers were placed on NMP at the donor centre. 300 patients were randomised which resulted in 151 transplants with the OCS device and 142 SCS livers. There was increased utilisation of DCD livers with NMP, as seen in the OrganOx trial (51 % NMP, 25 % SCS). In an intention to treat analysis, the incidence of early allograft dysfunction was lower with NMP (18 % NMP, 31 % SCS), with histological differences to support the better preservation. There was also a reduction in ischaemic biliary complications at 6 months with NMP in this study (2.6 % NMP, 8.5 % SCS, *P* = 0.02).

### Prolonged NMP

In addition to these novel techniques, researchers have demonstrated that NMP may be continued over a sustained period of several days to allow the liver to recover from ischaemia and reperfusion, and be transplanted, demonstrating liver regeneration in the process.^86^ The modified machine included a dialysis module, and a more complete attempt to regulate the endocrine influences on the liver.^87^ With this the Zurich group has transplanted livers that have undergone several days of NMP.^88^^,^^89^

## Other preservation modalities

In this review, we have focussed on the more common preservation modalities in clinical use, but there has been extensive research into other techniques. These range from ischaemia-free liver preservation pioneered in China,^90^^,^^91^ subnormothermic perfusion,^92^^,^^93^ and cryopreservation of the whole liver.^94^, ^95^, ^96^, ^97^ Each offers different opportunities but is some way from universal acceptance.

## Limitations of machine preservation

Machine perfusion offers many potential advantages, but instrumenting livers to undergo perfusion, and prolonged perfusion itself, are not without risks.

### Perfusate

While machine perfusion UW solution was developed for HMP of kidneys, it was not developed for oxygenated MP of livers. Similarly, the ideal perfusate for NMP has not been developed, with most NMP using red cells suspended in a plasma substitute, be that Gelofusine, human albumin Solution or Steen solution. While the absence of neutrophils and platelets would be expected to reduce reperfusion injury, in reality the liver is a large repository of such cells which are activated at the start of perfusion.^98^^,^^99^

### Vascular Injury

There is a risk of damaging the vessels, particularly the hepatic artery and its intima, while cannulating the liver for perfusion, resulting in thrombosis post-transplant; thrombosis does not occur during NMP due to the presence of high doses of heparin.

### Exacerbating Ischaemic Injury

If the perfusion device fails there is a risk of further ischaemic damage, most marked if the liver was warm at the time (undergoing NMP or NRP). This is less of a concern with HMP or HOPE.

### Cradle Compression

Some machines are associated with complications peculiar to them, one of which is cradle compression. This is a consequence of resting the liver on its diaphragmatic surface during NMP which results in compression and non-perfusion of dependent segments.^100^ This problem is most common during NMP and arises in the first few minutes of perfusion when the vascular resistance is greatest and the weight of the liver increases the resistance to perfusion. During HOPE and HMP it is possible to add sufficient fluid to the liver container to add buoyancy and limit dependent compression.

### Infection

It is well-recognised that donor organs run the risk of carrying infection from donor to liver,^101^ or may simply harbour potentially infectious agents.^102^ Blood is an excellent culture medium and NMP with a blood based perfusate is an opportunity for micro-organisms to infect the liver. While addition of antibiotics may reduce this risk, inappropriate antibiotic selection will not prevent it.^103^^,^^104^ NMP is not alone in carrying this risk, and serious infection has been recorded in livers undergoing HOPE after a period of NRP.^105^

## Indications for machine perfusion

### Viability Assessment

The ability to assess viability using machine perfusion is one of its great attractions, since it minimises uncertainty and permits transplantation of livers that may otherwise have been discarded. Case reports, small series, and a clinical trial attest to its efficacy in this setting.^83^^,^^106^, ^107^, ^108^, ^109^, ^110^ Different criteria have been used for different perfusion techniques, but share similar principles in determining the degree of hepatocyte damage and function.

#### NRP

During NRP, viability has been assessed in terms of lactate metabolism and transaminase release, the latter reflecting hepatocyte necrosis and consequent loss of function.

#### NMP

Transaminase release and lactate metabolism have also been suggested to be important criteria determining NMP viability,^111^^,^^112^ with more emphasis being placed by most on the ability of the liver to metabolise lactate.^109^^,^^113^ This is in spite of the fact that few functioning hepatocytes are required to clear lactate from a small circuit volume. The reality is probably that damaged hepatocytes are making lactate and hence apparent clearance is a balance between functioning and damaged cells. Other metabolic criteria include the ability of the liver to incorporate glucose into glycogen, a function of zone 3 hepatocytes in contrast to lactate metabolism which is a function of zone 1 hepatocytes. In contrast, the ability to maintain pH requires function of both zone 1 and zone 3.^114^

The other important marker of viability in NMP is the initial glucose.^115^ As discussed at the outset, ATP generation in ischaemic conditions requires glucose. Depletion of glucose results in depletion of ATP and cell loss. A glucose measured just 15 min after the start of NMP that is in the “normal” range is abnormal in a liver undergoing NMP – in the Cambridge series this occurred in just 10 % of livers, and 10 % of the ones that were transplanted resulted in primary non-function.

Appearance and vascular flows have also been suggested to be important,^109^ but non-perfused liver lobes or segments, or poor portal or hepatic artery flows, usually result in disturbed biochemistry.

Bile chemistry has been suggested by ourselves, and echoed by others, as a way to determine cholangiocyte viability,^114^, ^115^, ^116^, ^117^ since “normal” bile is characterised by a high pH and low glucose relative to perfusate as a result of secretory and absorptive processes by cholangiocytes. However, while it may be able to identify livers very likely to develop cholangiopathy, the use of such markers is associated with a high false negative rate,^112^ reflecting the often patchy nature of cholangiopathy and the ability of non-affected cholangiocytes to produce “normal” bile chemistry. In addition, bile chemistry cannot reflect proximal bile duct injury which may cause first and second order duct strictures.

#### HOPE

There is no near-patient functional assessment of livers undergoing HOPE, but instead the degree of mitochondrial injury has been shown to be a surrogate of functional capacity post-transplant.^51^ Microdialysis of the liver during HOPE has been suggested to provide some functional assessment in a small series of 10 perfusions.^118^

### Donation After Circulatory Death Livers

In addition to NRP becoming the recovery technique of choice for DCD livers and other organs, preserving organs using a perfusion technology is desirable, particularly where NRP is not used.

### Extended Preservation

All perfusion techniques involving oxygen administration appear to be able to extend liver preservation. Commencing perfusion at the donor hospital is optimal; commencing it in the recipient centre risks the liver exhausting its glucose supply during cold storage, and consequentially depleting ATP, resulting in cell death. Subsequent restoration of oxygen with NMP or HOPE will not resurrect function. This is a particular concern with DCD livers, which have undergone a period of warm ischaemia depleting energy stores and glucose stores.

### Steatotic Livers

One of the opportunities that ESMP offers is in the management of steatotic livers. One strategy is to place the liver on NMP immediately in the donor hospital, and so avoid cooling the liver down and suffering the enhanced reperfusion injury that characterises steatotic livers. Alternatively, ischaemia-free preservation would avoid any cooling, even during organ extraction.^90^

Proponents of HOPE suggest that reperfusion injury with the lower oxygen levels results in much less reperfusion injury; the same is probably true of COR. It is probable that NMP devices will need to change their algorithm to minimise early oxygenation and adopt slow rewarming in order to avoid severe reperfusion injury at normothermia.

Some authors have suggested de-fatting a liver during NMP would be beneficial,^119^, ^120^, ^121^, ^122^ but one of the main problems with steatotic livers is the reperfusion injury,^123^ and once established on NMP de-fatting may have little to offer. During long periods of NMP the fat content of livers reduces,^89^ as it does after implantation in the absence of ESMP.^124^

### Infection

While machine perfusion carries a risk of infection, it may permit the identification of infection from a donor and facilitate targeted treatment,^125^ including treatment of multi-resistant bacteria.^126^ Isolated liver perfusion also offers the possibility of using potentially toxic levels of antimicrobial agents, or antimicrobial agents toxic to other organs but not the liver, in order to achieve a therapeutic effect. There may also be an opportunity to treat livers infected with viruses with high dose combination antiviral agents, or with agents designed to prevent viral replication. One such target is a small interfering RNA (siRNA) against micro-RNA-122, a critical factor in HCV replication. It has been shown that Miravirsen, which sequesters miR-122, suppresses HCV replication during ESMP, and that its uptake was better during NMP than HMP.^127^

### Intrahepatic Microthrombi

It is becoming apparent that organs may accumulate intravascular fibrin *de novo* during ischaemia,^128^^,^^129^ and that this may result in cholangiopathy and sinusoidal obstruction.^130^^,^^131^ This is particularly troublesome in DCD livers, and those with prolonged periods of cold ischaemia. While strategies to inject recombinant tissue plasminogen activator (TPA) into the artery at the time of implantation have been utilised to reduce the incidence of cholangiopathy,^132^^,^^133^ this runs the risk of exacerbating post-implant bleeding. The other benefit of TPA is in the removal of sinusoidal fibrin, reducing vascular resistance.^131^ If TPA is administered during NMP, it needs a source of plasminogen such as fresh frozen plasma or plasminogen itself, since plasmin is the active component.^134^ Direct infusion of plasmin may be the preferred agent were it available.^134^

During hypothermic perfusion fibrin will not be cleaved, but oxygenation may prevent its formation and there may also be a mechanical effect of flushing the vasculature in clearing fibrin.^130^ However, this does not appear to be completely effective, as witnessed by the persisting incidence of non-anastomotic strictures following D-HOPE.^52^

### Other Interventions

The ability to perfuse an organ *ex situ* offers the opportunity for many other interventions. It has already been shown possible to split a liver *ex situ* while undergoing machine perfusion.^135^, ^136^, ^137^ Cellular therapies have also been suggested, such as infusion of mesenchymal stem cells with many different aims.^138^ Other cellular therapies include bile duct organoids to repair bile ducts.^139^ ESMP may permit modifying the immunogenicity of the liver, and recent work in kidneys has suggested that stripping blood group antigens from endothelial cells may permit transplantation across ABO differences.

## Summary

The landscape of liver transplantation is changing, and the opportunities offered by machine perfusion are set to make more of the currently available organs fit for transplantation, as well as making more organs available. Technology is in its infancy at present, and will improve over the next decade. While some livers are unlikely to benefit significantly, such as those from young fit donors implanted with minimal ischaemia, it is likely that machine perfusion will become commonly applied either in the form of NRP at retrieval for DCD livers, or ESMP post extraction for DBD and DCD livers. There is also the opportunity of combining technologies to allow their individual strengths to work synergistically. This is commonly done for DCD donation in Italy where the mandated long asystolic time results in significantly compromised livers.^140^

## CREDIT AUTHORSHIP CONTRIBUTION STATEMENT

Christopher Watson: Investigation, writing original draft; visualisation.

Rohit Gaurav: Investigation; writing review and editing.

Andrew Butler: Investigation; writing review and editing.

## Conflicts of interest

CJEW has received honoraria for lectures from OrganOx Ltd.

AJB has shares in a patent for the OrganOx *metra*.

RG has no conflicts of interest to declare.

## Funding

This work is part funded by the National Institute for Health and Care Research (NIHR) Blood and Transplant Research Unit in Organ Donation and Transplantation (NIHR203332), a partnership between NHS Blood and Transplant, University of Cambridge and Newcastle University, and part by the NIHR Cambridge Biomedical Research Centre (NIHR203312). The views expressed are those of the authors and not necessarily those of the NIHR, NHS Blood and Transplant or the Department of Health and Social Care.

The University of Cambridge has received salary support in respect of CJEW from the NHS in the East of England through the Clinical Academic Reserve.
